# Functional Outcomes in Surgically Treated Clavicle Fractures: A Longitudinal Observational Study

**DOI:** 10.7759/cureus.48081

**Published:** 2023-10-31

**Authors:** Prakhar Maheshwari, Chetan Peshin, Digvijay Agarwal

**Affiliations:** 1 Orthopedics, Royal Oldham Hospital, Manchester, GBR; 2 Orthopedic Surgery, Himalayan Institute of Medical Sciences, Dehradun, IND; 3 Orthopedics, Himalayan Institute of Medical Sciences, Dehradun, IND; 4 Orthopedics, Swami Rama Himalayan University, Dehradun, IND

**Keywords:** anatomical plates, radiological assessment, constant-murley score, plate fixation, operative fixation, clavicle fractures

## Abstract

Introduction

The clavicle is the most unique long bone and has a significant incidence in terms of fractures. Operative fixation for clavicle fractures has seen a steep rise in terms of technique as well as type of implant. Although extensive studies have been carried out in relation to clavicle fractures and their treatment modalities, no proper guidelines or approach has been identified as ideal, and hence, this study was carried out to evaluate operative fixation as a viable strategy.

Objective

The objective of this article was to assess functional outcomes of plate fixation in clavicle fractures.

Materials and methods

This longitudinal prospective observational study included 30 patients treated for clavicle fractures with plate fixation in the Orthopedics Department of Himalayan Institute Hospital Trust (HIHT), Jollygrant, Dehradun, over a period of one year. Functional outcomes were assessed as Constant-Murley shoulder scores, and complications were recorded. Radiological assessment was done on the basis of time to union through follow-up skiagrams. Statistical analysis was performed using the SPSS statistical package version 17.0 (IBM Inc., Armonk, New York). Continuous variables are presented as mean ± SD, and categorical variables are presented as absolute numbers and percentages. Continuous variables and constant score values over time within the groups were analyzed using repeated measures analysis of variance (ANOVA) followed by Bonferroni's post hoc testing. A p-value of <0.05 was considered statistically significant.

Results

The mean age of patients undergoing surgical fixation of clavicle fractures was 36 ± 12.53 years, ranging from 18-65 years. Of the entire study group, 83.3% were males and 16.7% were females. Road traffic accident (RTA) was the most common cause of clavicle fracture, constituting 76.7% of the entire study population, followed by fall on the floor (20.0%), and one patient sustained trauma by being hit by a bull (3.3%). Our study demonstrated a mean Constant score of 73.87 ± 2.64, 82.80 ± 2.20, and 92.40 ± 2.37 at one-month, two-month, and four-month follow-up times, respectively, which was found to be statistically significant in terms of progression (p value<0.001). The mean union time of clavicle fractures in our study population was 12.1 weeks. Two patients in our study developed implant impingement.

Conclusion

Our study revealed that patients with clavicle fractures treated with plate fixation had statistically significant good functional outcome (Constant) scores at sequential follow-ups, consistent with available literature. Mean union time was also comparable to existing literature. Non-union was not noted in our study, and only two cases developed implant impingement. Hence, we conclude that early primary plate fixation for displaced clavicle fractures is a promising technique with good overall functional outcomes and patient satisfaction, especially in young, active patients.

## Introduction

The clavicle is one of the most unique long bones with many special characteristics, including its shape and geometry, which makes it prone to getting fractured. Clavicle fractures are among the most common skeletal injuries, accounting for 2-5% of all adult fractures, with an incidence of 29-64 cases per 100,000 [1,2]. The clavicle, owing to its position and subcutaneous location, is susceptible to trauma both due to direct blows as well as due to fall on an outstretched hand. Fractures of the clavicle might even result due to metastatic and metabolic conditions. Approximately 69-81% of clavicle fractures are in the middle one-third of the clavicle, which is weaker as compared to other parts of the clavicle and contains sparse soft tissue. Seventeen percent of clavicle fractures are in the lateral one-third, and 2% are in the medial one-third [3].

Traditionally, fractures of this bone, connecting the shoulder girdle to the sternum, were treated conservatively with appropriate splinting and rest, but throughout the decade, the operative fixation rates for clavicular fractures have shown an increasing trend. Conservative approaches with the use of chest arm immobilizer/ shoulder arm pouches as well as bandaging in a figure of eight have been widely used with successful outcomes in many cases. Operative fixations using an intramedullary approach with wires as well as plating have seen a steep rise. Plate fixation of clavicular fractures has advanced in approach, starting from reconstruction plates to non-anatomic plates while finally arriving at pre-contoured anatomical plates. The plates can even differ in material, ranging from stainless steel to titanium.

The major complications of clavicle fractures include non-unions, malunions, neurovascular injury, and injury to mediastinal structures, along with future cosmetic drawbacks.

The aim of clavicle fracture treatment is primarily obtaining a good shoulder function and avoiding the aforementioned complications. Shoulder function is a parameter that has often been neglected when outcomes of clavicle fracture are described but, in essence, is one of the most significant aspects related to patient satisfaction and physician goals. No definitive guidelines have been set to demarcate and decide the choice of treating clavicle fractures through conservative or operative approaches. Moreover, non-operative treatment has been challenged by the increasing popularity and rate of surgical fixations in recent years despite a lack of clear evidence in the current literature [4]. However, surgery should be considered for open fractures, compromised skin conditions, neurological deficiencies, vascular injury, ipsilateral serial rib fractures, or floating shoulder [5].

Conservative management of clavicle fractures in the majority results in union and a positive outcome. However, many studies have been done to compare the two approaches. The parameters to differentiate the approaches are majorly based on non-union rate, malunion rate, rate of complication, post-treatment shoulder function, shortening, post-treatment cosmetic outcome, time duration required for immobilization, and radiographic differences in healing and alignment. Various scoring systems like the DASH score and Constant-Murley scores have been used widely to assess shoulder function before or after treatment of clavicle fractures.

Although extensive studies have been carried out in relation to clavicle fractures and their treatment modalities, no proper guidelines or approach has been identified as ideal, and the treatment is usually tailor-made. This study aims to assess the functional outcome of clavicle fractures treated with plate fixation in a prospective approach using Constant-Murley scoring.

## Materials and methods

The prospective longitudinal observational study was conducted at the Himalayan Institute of Medical Sciences (HIMS), Swami Ram Nagar, Dehradun, over a period of 12 months, starting from 1st January 2020 to 31st December 2020.

Patients with acute displaced clavicle fractures admitted to the Himalayan Institute of Medical Sciences who met the inclusion criteria and operated with plate fixation were incorporated into the study.

Informed written consent was taken from each patient before including them in the study.

Ethical clearance was also taken from the institute ethics committee (ECR/483/Inst/UK/2-13/RR-16, Dt. 23.8.2017).

Patients were treated with anatomic pre-contoured locking compression plates using the usual incision and placing the plate over the superolateral surface of the clavicle.

Study design

**Figure 1:**
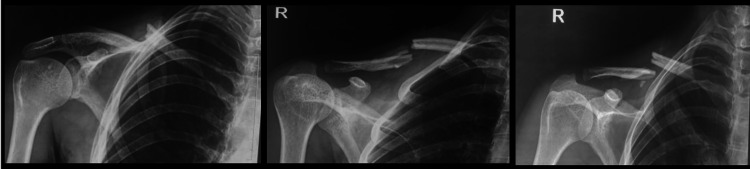
Displaced clavicle fracture - AP, cephalic, and caudal X-ray views

Our study was a prospective longitudinal observational study including 30 patients above 18 years of age with traumatic clavicle fractures operated within two weeks of trauma with plate fixation at the Department of Orthopaedics, HIMS, SRHU from 1st January 2020 to 31st December 2020. Patients with pathological fractures, bilateral fractures, fractures due to metabolic diseases, and patients with pre-existing shoulder pathologies like periarthritis, rotator cuff disease, etc., were excluded from the study. Patients were admitted with suspected clavicle fractures and underwent X-rays in AP/cephalic/caudal views to assess the type of fracture. Operated patients were followed up at one month, three months, and six months (or till bony union was achieved), along with repeat X-rays. X-ray images of a displaced clavicle fracture with multiple views are shown in Figure 1. 

After obtaining consent from each subject, subjects were scored using the Constant-Murley Score [6] at the follow-up points.

**Figure 2:**
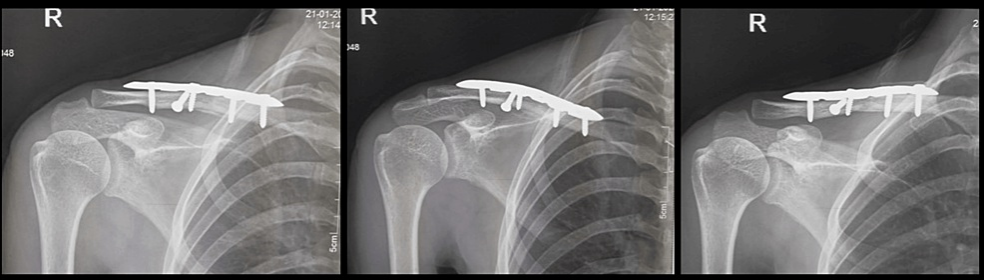
Post-operative clavicle fracture - AP, cephalic, and caudal X-ray views

Patients were treated with anatomic pre-contoured locking compression plates using the usual incision and placing the plate over the superolateral surface of the clavicle. The post-operative X-rays with the plate are shown in Figure 2.

**Figure 3:**
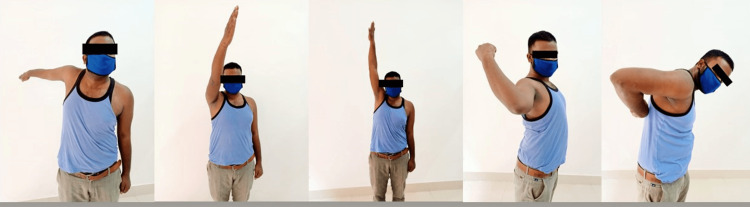
Range of movement at shoulder joint post-operatively

The functional score (Constant-Murley Score) of patients was calculated post-operatively, which also included a range of motion measurement at the shoulder joint, as shown in Figure 3.

Statistical analysis was done on the data obtained. Constant-Murley score is a functional assessment score for upper limb conditions that can be used pre-operatively and post-operatively. It is a mixed score with objective as well as subjective assessment parts. The maximum score is 100, with a numerical score nearer to 100 judged as a better score in terms of function. Statistical analysis was performed using the SPSS statistical package version 17.0 (IBM Inc., Armonk, New York). Continuous variables are presented as mean ± SD, and categorical variables are presented as absolute numbers and percentages. Continuous variables and constant score values over time within the groups were analyzed using repeated measures analysis of variance (ANOVA) followed by Bonferroni's post hoc testing. A p-value of <0.05 was considered statistically significant. 

## Results

A total of 30 patients were included in the study. All patients included in the study underwent plate fixation for clavicle fracture.

In this study, the mean age of patients who underwent surgical fixation of clavicle fractures was 36.1 ± 12.5 years, ranging from 18-65 years.

On analyzing the age distribution among patients, it was observed that this type of injury was more common in young adults and less in the elderly, as shown in Table 1.

**Table 1:** Age distribution among patients (n=30)

Age groups	Frequency	%
16 - 30 yrs	13	43.4%
31 - 45 yrs	10	33.3%
>45 yrs	7	23.3%
Total	30	100%

According to this study, males were more prone to sustain clavicle fractures as compared to females. Of the entire study, 83.3% were males, and 16.7% were females. The distribution of the mode of injury pattern in this study is shown in Table 2.

**Table 2:** Association with mode of injury (n=30) RTA - road traffic accident

Mode of trauma	Frequency	%
Fall on floor	7	16.3%
Hit by bull	1	2.3%
RTA	34	79.1%
Slip on floor	1	2.3%
Total	43	100%

RTA was the most common cause of clavicle fracture, constituting 76.7% of the entire study population, followed by falls on the floor (20.0%), and one patient sustained trauma by being hit by a bull (3.3%). Constant score distribution with respect to follow-up times is described in Table 3.

**Table 3:** Constant score at follow-up intervals (n=30)

Constant score	N	Mean ± SD	p-value
1 month	30	73.87 ± 2.64	<0.001
3 months	30	82.80 ± 2.20	
6 months	30	92.40 ± 2.37	

This study demonstrated a mean constant score of 73.87 ± 2.64, 82.80 ± 2.20 & 92.40 ± 2.37 at one month, three months, six months follow-up times, respectively, which was found to be statistically significant in terms of progression (p-value<0.001). The mean union time of clavicle fractures in the study population was 12.1 weeks, with the distribution shown in Table 4.

**Table 4:** Distribution of radiographic union time (weeks) (n=30)

Radiographic union time (weeks)	%
10 - 12 weeks	63.3%
12 - 16 weeks	36.7%

## Discussion

In spite of extensive studies in relation to clavicle fractures and its treatment modalities, to date, no proper guidelines or approach has been identified as ideal. In the past, displaced clavicle fractures were treated conservatively with good results. This was faced with criticism in many studies that were carried out in terms of non-unions as well as malunions. Conservative treatment often led to clavicular shortening and decreased shoulder function in the long term and asymmetry of the shoulder.

In due course of time, patient satisfaction became an important element in the functional outcome, and the demands of patients grew. Gradually, studies were carried out with operative fixation of clavicle fractures using intramedullary nailing implants, K wires, and Knowles pin, but they did not provide appropriate length and rotational stability.

Plating was introduced to tackle these shortcomings, and it provided immediate rigid fixation, helping to facilitate early mobilization. Further advancement in implant knowledge and biomechanics led to the advent of pre-contoured anatomical locking clavicle plates, which gave even better results. With improved implants, prophylactic antibiotics, as well as better soft tissue handling, plate fixation became a reliable and reproducible technique. Operative management with plating has provided a better functional outcome. It has significantly reduced malunion but has also given rise to surgery and implant-related complications, which pose a challenge to the efficacy of operative management.

Complications like implant impingement, infection, hypertrophic scarring, neurovascular injury, hardware migration, etc., are associated with surgical fixation of clavicle fractures, even though their incidence might vary.

In this study, we have tried to make an ardent attempt to assess functional outcomes of clavicle fracture plate fixation in terms of Constant-Murley scores at one-month, three-month, and six-month follow-ups, along with a radiological assessment of union. Thirty subjects, which fulfilled the inclusion criteria, were taken into consideration. None of the patients were lost to follow-up. The data obtained was subjected to statistical analysis and was compared with existing literature to the best of our knowledge.

A pattern of age distribution in our study showed that clavicle fracture is more common in young adults and was found to be in accord with different other studies as compared in Table 5.

**Table 5:** Comparison of age distribution with literature

Study	Number of patients	Mean age (years)
Canadian Orthopaedic Trauma Society (COTS) [7]	132	33.5
Byron Chalidis et al. [8]	139	39.3
Daniilidis et al. [9]	151	40.3
Robinson et al. [10]	200	32.3
Van Der et al. [11]	97	40.8
Woltz et al. [12]	160	38.3
Verborgt et al. [13]	39	28
Naveen et al. [14]	60	32.4
Our study	30	36.13

Further, in our study, we found that males are way more prone to sustain clavicle fractures as compared to females. A similar trend was seen in many studies compared to Table 6.

**Table 6:** Comparison of gender distribution with literature

Study	Number of patients	Male	Female
Chalidis et al. [8]	139	105	34
Faldini et al. [15]	100	78	22
De Giorgi et al. [16]	71	51	20
Daniilidis et al. [9]	151	115	36
Robinson et al. [10]	200	175	25
Verborgt et al. [13]	39	34	5
Our study	30	25	5

In our study population, MVA was the most common mode of injury, and a similar trend was observed in other studies that were compared in Table 7.

**Table 7:** Comparison of the mode of injury between the current study and available literature RTA - road traffic accident

Study	Mode
Kulshrestha et al. [17]	RTA- 23 (51%)
	Assault - 3 (7%)
	Sports - 7 (15%)
	Fall - 12 (27%)
Van Der et al. [11]	RTA - 30 (76%)
	Fall - 1 (3%)
	Sports- 8 (21%)
Robinson et al. [10]	RTA - 34
	Fall - 22
	Sports - 39
Canadian Orthopaedic Trauma Society (COTS) [7]	RTA - 42
	Fall - 9
	Sports - 9
Our study	RTA - 23
	Hit by bull - 1
	Fall - 6

The mean union time in our study was 12.1 weeks, which was comparable to the available literature shown in Table 8.

**Table 8:** Comparison of mean union time with available literature

Study	Operative
Canadian Orthopaedic Trauma Society (COTS) [7]	16.4
Nandi et al. [18]	14.6
Dugar et al. [9]	15.73
Verborgt et al. [13]	15
Mohammed et al. [19]	14
Naveen et al. [14]	9.27
Our study	12.1

Non-union is a very significant entity governing the functional outcome and satisfaction of the patient. Hence, we have compared non-union status in our study with the available literature in Table 9.

**Table 9:** Comparison of non-union between current study and available literature

Study	Non-union (No. of cases)
Canadian Orthopaedic Trauma Society (COTS) [7]	2
Kulshrestha et al. [17]	0
Virtanen et al. [20]	0
Daniilidis et al. [9]	0
Robinson et al. [10]	1
Van Der et al. [11]	1
Woltz et al. [12]	9
Qvist et al. [21]	2
Our study	0

Our study demonstrated a statistically significant progressive constant score at all follow-up points and a good functional outcome in all patients. A comparison of different studies available in the literature is depicted in Table 10.

**Table 10:** Comparison of functional outcome score in the available literature

Study	Constant score			
	6 Weeks	3 Months	6 Months	1 Year
Robinson et al. [10]	70			92+- 9.5
Canadian Orthopaedic Trauma Society (COTS) [7]	80	90	94	96+-5
Virtanen et al. [20]				86.5+- 11.5
Woltz et al. [12]				95.4 +- 7.8
Melean et al. [22]				93 +- 1
Mirzatolooei et al. [23]				89.8 +- 6
Verborgt et al. [13]	88 (50-98)			
Van Der et al. [11]	90.9 +- 14.2		95.9 +- 10.5	
Daniilidis et al. [9]				91.7
Mohammed et al. [19]				95.33 +- 3.4
Naveen et al. [14]	71.80 +- 4.87	83.63 +-4.82	94 +- 2.99	
Nandi et al. [18]				96.8
Qvist et al. [21]	90	97	98	98

A comparison of functional outcome scores in our study is depicted in Table 11.

**Table 11:** Comparison of functional outcome score in our study

	1 Month	3 Months	6 Months
Our study	73.87 ± 2.64	82.80 ± 2.20	92.40 ± 2.37

The limitations of our study include a relatively small sample size of the study population and lack of a comparative conservatively treated group. A longer follow-up would also have been desirable to assess functional scores in the long term better and to note any other complications in the operative treatment strategy.

## Conclusions

We found that patients with displaced clavicle fractures treated with plate fixation had good functional outcome scores at sequential follow-ups. A Constant-Murley score was chosen as it combines objective as well as subjective components and hence proves to be a good functional outcome assessment tool.

In our study, the majority of clavicle fractures occurred in young adult males, with RTA being the predominant mechanism of injury. Post-operative functional scores found in our subjects were statistically significant in terms of p-value on analysis. Hence, we conclude that early primary plate fixation for displaced clavicle fractures is a promising technique with good overall functional outcomes and patient satisfaction, especially in young, active patients.
